# Imbalanced immune cell network and suboptimal cell activation: signatures associated with disease severity in vaccine-naïve COVID-19 patients

**DOI:** 10.3389/fimmu.2026.1794103

**Published:** 2026-03-10

**Authors:** Ranferi Ocaña-Guzman, Elvira Piten-Isidro, Julio Flores-Gonzalez, Lucero A. Ramon-Luing, Perla M. Del Rio-Estrada, Ramcés Falfán-Valencia, Gloria Pérez-Rubio, Ivette Buendia-Roldan, Moisés Selman, Leslie Chavez-Galan

**Affiliations:** 1 Laboratorio de Inmunología Integrativa, Instituto Nacional de Enfermedades Respiratorias Ismael Cosío Villegas, Mexico City, Mexico; 2 Departamento de Investigación en Enfermedades Infecciosas, Instituto Nacional de Enfermedades Respiratorias Ismael Cosío Villegas, Mexico City, Mexico; 3 Pathology Advanced Translational Research Unit (PATRU), Department of Pathology and Laboratory Medicine, Emory University School of Medicine, Atlanta, GA, United States; 4 Laboratorio de Neumogenómica, Instituto Nacional de Enfermedades Respiratorias Ismael Cosío Villegas, Mexico City, Mexico; 5 Clínica de Investigación Traslacional en Envejecimiento y Enfermedades Fibrosantes, Instituto Nacional de Enfermedades Respiratorias Ismael Cosío Villegas, Mexico City, Mexico; 6 Laboratorio de Biopatología Pulmonar INER-Ciencias-UNAM, Instituto Nacional de Enfermedades Respiratorias Ismael Cosío Villegas, Mexico City, Mexico

**Keywords:** cell activation, COVID-19, immune cells, severity, vaccine-naïve

## Abstract

**Background:**

COVID-19 vaccination has significantly reduced mortality and morbidity. Recent studies in unvaccinated people indicate a more complex immune response beyond just the cytokine storm. Understanding changes in the immune cell network is crucial for identifying vaccine-independent immune imbalances, especially in vaccine-naïve patients needing invasive mechanical ventilation (IMV). This knowledge could help improve vaccine development and find biomarkers linked to severe COVID-19.

**Methods:**

Peripheral blood immune cells from vaccine-naïve COVID-19 patients from the first pandemic wave were classified into those who required IMV and those who did not (No-IMV). High-dimensional immune phenotyping was performed using multiparametric flow cytometry combined with FlowSOM clustering and UMAP for dimensionality reduction. Additionally, T-cell activation efficiency after polyclonal stimulation was evaluated *in vitro*.

**Results:**

IMV patients, but not No-IMV, exhibited a marked disruption of immune cell networks, characterized by a loss of immune checkpoint (IC)-expressing T-cell subsets, particularly PD-1- and LAG-3-expressing T cells. Conversely, there was an increase in the frequency of T cells co-expressing molecules linked to inflammatory pathways (TNF/TNFR) and cell death (CD95L). These changes were also associated with reduced CD8^+^ T-cell activation capacity and the rise of non-conventional cytotoxic CD4^+^ T-cell subsets. In the B-cell compartment, IMV patients displayed depletion of CCR7+ subsets and decreased PD-1 expression. Additionally, higher frequencies of NK and NKT cells expressing TNF pathway-related molecules were observed. While classical monocyte subsets expressing ICs such as PD-L1, PD-L2, and TIM-3 remained stable, non-classical monocyte subsets showed altered IC expression. In contrast, No-IMV patients maintained a relatively balanced immune architecture.

**Conclusions:**

Vaccine-naïve COVID-19 patients requiring IMV display an immune landscape distinct from that of No-IMV patients. IMV exhibits a profound imbalance in innate and adaptive immune cell networks, characterized by inflammatory skewing, loss of regulatory subsets, and impaired cytotoxic T-cell functionality, features not observed in No-IMV. These findings reveal coordinated immune alterations beyond cytokine hyperinflammation and identify cellular immune signatures associated with severe COVID-19.

## 1 Introduction

COVID-19 is an infectious disease caused by the severe acute respiratory syndrome coronavirus 2 (SARS-CoV-2), which infects host cells through the interaction of its spike (S) protein with the angiotensin-converting enzyme 2 (ACE2) receptor (1). Since March 2020, COVID-19 has caused nearly 779 million confirmed cases worldwide, and following the emergency authorization and widespread use of anti-COVID-19 vaccines, both mortality and the incidence of severe disease have decreased substantially (2).

SARS-CoV-2 infection can induce a spectrum of symptoms, from asymptomatic to critical (3). Host-related factors such as advanced age or comorbidities significantly influence disease progression and outcomes (4). Since the first reports on the physiopathology of COVID-19, the phenomenon known as the “cytokine storm” has been considered a cornerstone of severe cases (5). However, more recent studies conducted in unvaccinated individuals have revealed a more complex immunopathology. Critically ill patients may exhibit either hyperactive or hypoactive immune responses, suggesting that severe COVID-19, particularly in those requiring invasive mechanical ventilation (IMV), can arise through distinct immunological alterations and even gene variants associated with cytokine and Toll-like receptor (TLR) have also been associated with poor clinical outcomes (6, 7).

Despite significant advances in therapeutic strategies and vaccination programs, critical questions remain regarding the mechanisms that drive disease severity. Numerous immune alterations have been described in severe COVID-19, including reductions in total T lymphocytes (CD4+ and CD8+ T cells), selective depletion of cytotoxic CD8+ T cells, and alterations in the lymphocyte-to-monocyte ratio (8). Studies from the first wave of the pandemic, before vaccine availability, reported increased infiltration and marked activation of immune cells, including macrophages and CD4+ and CD8+ T cells, in lung tissue. Likewise, at the systemic level, elevated soluble mediators, including tumor necrosis factor receptor 1 (TNFR1) and A disintegrin and metalloprotease 17 (ADAM17), were associated with fatal outcomes, reflecting a state of immune hyperactivation in critical patients, mainly those who died (9).

A balanced immune response requires the involvement of both activation and inhibition pathways. Inhibitory receptors (IR) or immune checkpoints (IC) regulate immune responses. Some of the better-characterized IRs include Programmed Cell Death-1 (PD-1), Cytotoxic T-Cell Lymphocyte-Associated protein-4 (CTLA-4), T cell Immunoglobulin and Mucin domain-3 (TIM-3), Lymphocyte-Activation Gene 3 (LAG-3), and Killer cell Lectin-like Receptor G1 (KLRG-1) (10).

ICs have attracted increasing attention as therapeutic targets in diseases characterized by immune dysregulation; however, blocking ICs should be approached with caution, as it could trigger uncontrolled immune reactivation (11). Several studies have documented dysfunctional CD8+PD-1+T cells in COVID-19 patients, with an association between PD-1 expression and elevated soluble PD-L2 levels. More recently, elevated PD-L2 concentrations at 12 months post-infection have been linked to persistent lung lesions, suggesting that excessive IC signaling may contribute not only to acute disease severity but also to long-term pulmonary sequelae (12, 13).

In this context, the present study aims to perform a comprehensive immune phenotypic characterization of vaccine-naïve patients from the first wave of the pandemic, stratified by the requirement for IMV as a marker of disease severity. This approach provides an opportunity to understand the vaccine-independent immune imbalance and refine our interpretation of immune phenotypes associated with severity, which may help identify biomarkers of severe COVID-19.

## 2 Methods

### 2.1 Study population

COVID-19 patients were recruited from May to December 2020 in the Instituto Nacional de Enfermedades Respiratorias Ismael Cosío Villegas (INER), in Mexico City. Diagnosis was made for clinicians based on combined clinical criteria and confirmed by RT-PCR of nasopharyngeal swabs. The study included 30 COVID-19 patients ≥60 years old without evidence of other pulmonary infectious or chronic diseases. Groups displayed a similar frequency of known risk factors for severity, such as diabetes, hypertension, and smoking (**Table 1**). In addition, a control group of healthy donors (HD, n=10) was included; this group was matched for age and sex, and all HD were negative for SARS-CoV-2 and clinically qualified as healthy subjects. All samples, including HD, were obtained before the pandemic because they were from the aging cohort of our Institute.

**Table 1:** Demographic data of the study subjects.

Demographic data	No-IMV n = 10	IMV n = 20	HD n= 10	p-value
*Age (+/- SD)*	51 (20.5)	54 (11.4)	65 (4.1)	NS
*Sex, male (%)*	9 (90)	9(45)	3 (30)	NS
*BMI (+/- SD)*	28(2.4)	35 (4.3)	26 (4.5)	NS
*Smoking (%)*	5(50)	5(25)	6 (60)	NS
*Hypertension (%)*	5(50)	5(25)	4 (40)	NS
*Diabetes (%)*	3(30)	4(20)	2 (20)	NS
*Cough(%)*	4(40)	14(70)	NA	NS
*Dyspnea(%)*	5(50)	18(90)	NA	NS
*Myalgias(%)*	5(50)	13(65)	NA	NS
*Arthralgias(%)*	4(40)	12(60)	NA	NS
*Headache(%)*	4(40)	13(65)	NA	NS
*Rhinorrhea(%)*	0	9(45)	NA	NS
*Diarrhea(%)*	0	9(45)	NA	NS
*Anosmia(%)*	1(10)	10(50)	NA	NS
*Ageusia(%)*	2(20)	9(45)	NA	NS
*PAFI (+/- SD)*	213(37.7)	130 (28.8)	NA	<0.0001

Continuous values are presented as mean (standard deviation), and categorical data are presented as frequencies and percentages. BMI, body mass index; PAFI, PaO_2_/FIO_2_ ratio; NA, not apply; NS, no statistical differences; No-IMV, COVID-19 patients without invasive mechanical ventilation; IMV, COVID-19 patients who received invasive mechanical ventilation. HD, healthy donors. Brown-Forsythe test/ANOVA, χ2 test.

Clinical, sociodemographic, and epidemiological data for each patient were collected at admission and stored in the institutional electronic records; these data were subsequently retrieved from that system.

### 2.2 Severity classification of COVID-19

Patients were divided into two groups: those who did not receive invasive mechanical ventilation (No-IMV), who mostly presented with moderate disease severity, and those who required invasive mechanical ventilation (IMV), most of whom had severe or critical condition.

A chest CT scan was used to assess for pneumonia, and the PaO2/FiO2 ratio (PAFI) was used to assess disease severity (severe: 199-125; critical: <120).

### 2.3 Ethical approval

The Institutional Review Board of the INER in Mexico City revised and approved the current investigation (#C41-20, B23-23). All patients provided written informed consent to participate in the study, and all procedures were performed in accordance with the 1964 Helsinki Declaration and its most recent version.

### 2.4 Peripheral blood mononuclear cells

A blood sample was obtained within 4 days of hospital admission. Then, peripheral blood mononuclear cells (PBMCs) were isolated from 20 mL of whole heparinized blood by ficoll density gradient (Lymphoprep™ Axis-Shield, Oslo, Norway). The PBMC interface was washed with phosphate-buffered saline (PBS 1X), and the cells were resuspended in NutriFreez D10 (Sartorius, Göttingen, Germany) cryopreservation medium containing 10% dimethyl sulfoxide and stored in liquid nitrogen until use. Plasma was aliquoted and stored at -20 °C until use.

### 2.5 Cell culture and *in vitro* stimulation

Cells were unfrosted, washed with 1X PBS, and counted using a TC20™ automated cell counter (Bio-Rad, Hercules, CA, USA). Trypan blue exclusion confirmed a minimum of 85% viability to use the cells.

Then, cells were plated at 1 × 10^6^ cells/well in 24-well plates (Sarsted, Germany) with RPMI 1640 medium (Sigma-Aldrich, Missouri, USA) supplemented with L-glutamine (2mM; GIBCO, Grand Island, NY, USA), streptomycin-penicillin mix solution (Sigma-Aldrich, Missouri, USA), and 10% heat-inactivated fetal bovine serum (GIBCO, Grand Island, NY, USA). Cells were stimulated with phorbol-12-myristate-13-acetate (PMA) at 50 ng/mL and ionomycin (Iono) at 1 μg/mL for 6 hours. To block cytokine transport processes after cell activation, monensin solution (BioLegend, San Jose, CA) was added 5 hours before the end of the culture. The cells were collected and washed for flow cytometry analysis.

### 2.6 Multiparametric flow cytometry

We evaluated cell-surface marker expression on PBMCs using fluorochrome-conjugated monoclonal antibodies (mAbs) targeting diverse molecules. The complete list of mAbs used in this study is in **Supplementary Table 1**.

PBMCs were incubated with mAbs for 20 min at 4 °C in a staining buffer (BioLegend, San Jose, CA) for FACS analysis. Then, cells were washed and finally fixed with 4% PBS-paraformaldehyde.

For intracellular evaluation, fixation and permeabilization were performed using the Cytofix/Cytoperm™ Plus (BD, Franklin Lakes, NJ, USA) according to the manufacturer’s instructions.

To exclude dead cells, the side-scatter/forward-scatter gating strategy and a viability marker were used. A fluorescence minus one (FMO) control was employed to set the gates for specific immune cell subpopulations. At least 300,000 events per sample were acquired in a BD FACS Symphony A3 Cell Analyzer (Becton Dickinson, San Jose, CA). The compensation setup and the specific cell subset frequency calculation were performed using FlowJo (FlowJo, LLC, Ashland, OR).

### 2.7 Analysis of flow cytometry data

Compensated Flow Cytometry Standard (FCS) 3.0 files were imported into FlowJo software version v10.7.1 and analyzed by standard gating to remove anomalies based on the flow rate, signal acquisition, and dynamic range in each FCS data and select good events using FlowAI analysis (R package FlowAI, version 1.28.0) as a quality control analysis (**Supplementary Figure 1A**), remove doublets, aggregates, and dead cells (**Supplementary Figure 1B**). Next, a pre-gating to identify T-cells (CD3+), B-cells (CD3-CD20+) (**Supplementary Figure 1C**), NKT-cells (CD3+CD56+), NK (CD3-CD56+), monocytes (CD3-HLADR+CD14+), and CD3+ monocytes (CD2-CD3+CD14+) was done (**Supplementary Figures 2A–C**) to realize a concatenated file of each population (**Supplementary Figures 1D**, **Supplementary Figure S2D**). Clustering and dimensional reduction were performed using FlowSOM (version 2.4.0) and Uniform Manifold Approximation and Projection (UMAP) (version 0.2.8.0) algorithms, respectively (**Supplementary Figures 1E**, **Supplementary Figure S2E**).

The T or B-cell clusters were reanalyzed after the clustering using the following markers: LAG3, CD119, PD-1, CD27, CD45RA, PD-L1, CD95 (FAS), CD95-L (FAS-L), TNF-RI, TNF-RII, KLRG1, TNF, TIM-3, CCR7, and PD-L2 (**Supplementary Figures 3A, C**).

The NKT cell clusters were reanalyzed after the clustering using the following marks: CD2, TNF-RI, TNF, CD3, CD27, PD-L2, TIM-3, CD56, HLA-DR, CR-Va4-Ja18, TNF-RII, CD11b, PD-L1, and CD16 (**Supplementary Figure 4A**). For NK cell cluster analysis, the following marks were used: CD2, TNF-RI, TNF, CD27, PD-L2, TIM-3, CD56, HLA-DR, TNF-RII, CD11b, PD-L1, and CD16c (**Supplementary Figure 4C**).

To monocytes cells, TNF-RI, TNF, CD27, PD-L2, TIM-3, CD14, HLA-DR, TNF-RII, CD11b, PD-L1, CD16 were using as markers (**Supplementary Figure 5A**). Finally, monocytes and CD3+ monocyte clusters were reanalyzed using the following marks: TNF-RI, TNF, CD3, CD27, PD-L2, TIM-3, CD14, HLA-DR, CR-Va4-Ja18, TNF-RII, CD11b, PD-L1, and CD16 (**Supplementary Figure 5C**).

At the end of the T-cell stimulation with PMA/Iono, cells were prepared for flow cytometry, and a similar analysis was performed on CD3+ events from unstimulated and stimulated conditions in all groups. The CD4+ or CD8+ cell clusters were reanalyzed after the clustering using the following marks: CD27, IL-2, IFN-γ, TNF, Perforin (Perf), and Granzyme B (GrzmB) (**Supplementary Figure 6**).

### 2.8 Statistical analysis

Posterior to normality analysis, data are reported as medians with interquartile ranges (IQR 25^th^-75^th^ percentiles, 95% CI) for nonparametric distributions, and for parametric distributions, quantitative variables are reported as means with standard deviations. Categorical variables are shown with frequencies and percentages. Comparisons between 2 groups were evaluated with the U Mann–Whitney test or the Wilcoxon test, whereas multiple comparisons were performed with the Kruskal–Wallis test and corrected using Dunn’s test. (GraphPad Prism 9.0.1, Software, Inc., San Diego, CA, USA).

## 3 Results

### 3.1 Clinical and demographic characteristics of the study groups

This study included 30 patients with COVID-19, 20 who required invasive mechanical ventilation (IMV) and 10 who did not (No-IMV), as well as 10 healthy donors (HD) included as a control group. The groups showed similar demographic characteristics, with no significant differences, including risk factors associated with severity, except for the PaO_2_/FiO_2_ ratio (PAFI), which, as expected, was more severely impaired in the IMV group (**Table 1**). Biochemical parameters were generally comparable among groups; however, C-reactive protein levels were significantly higher in IMV patients compared with both No-IMV patients and HD (p < 0.0001) (**Table 2**).

**Table 2:** Biochemical data of the study subjects.

Biochemical parameter	No-IMV n = 10	IMV n = 20	HD n= 10	p-value
*Leukocytes cells/mL(± SD)*	10.6 (3.1)	13 (6.5)	6.13 (1.9)	NS
*Lymphocytes cells/mL(± SD)*	0.99 (0.4)	0.81 (0.6)	0.96 (0.8)	NS
*Platelets cells/mL(± SD)*	247.2 (68)	236.7 (88.4)	225.7 (49.6)	NS
*LDH UI/L(± SD)*	288.5 (67)	558 (222)	178 (30)	NS
*DD ng/mL(± SD)*	1.0 (0.8)	4.8 (3)	NA	NS
*Procalcitonin ng/mL(± SD)*	0.4 (0.3)	1.2 (1)	NA	NS
*Fibrinogen mg/dL(± SD)*	565.3 (150)	866 (169)	NA	NS
*PCR mg/dL(± SD)*	14.0 (4.1)	21.0 (9)	0.18 (0.12)	<0.0001

Continuous values are presented as mean (standard deviation). DHL, lactic dehydrogenase; DD, D-dimer; ESR, erythrocyte sedimentation rate; PCR, C-reactive protein; NA, not apply; NS, no statistical differences; No-IMV, COVID-19 patients without invasive mechanical ventilation; IMV, COVID-19 patients who received invasive mechanical ventilation. HD, healthy donors. Brown-Forsythe/ANOVA test.

### 3.2 Altered T-cell frequency and inflammatory profile in IMV COVID-19 patients

**Figure 1:**
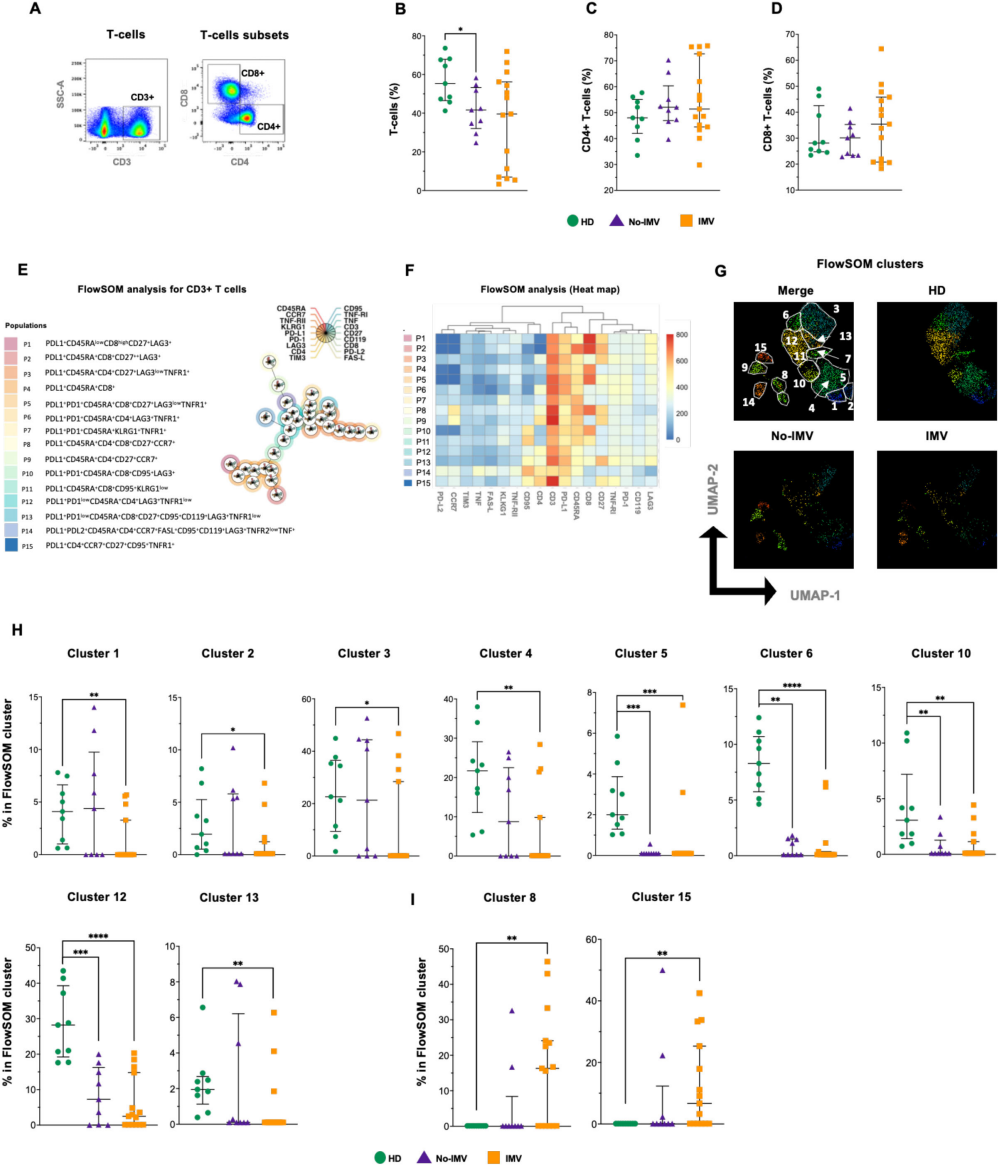
High-dimensional cytometry data reveal differences in the distribution of T-cell subpopulations of COVID-19 patients. Representative dot plots showing manual gating analysis of total T-cells (CD3+) and their subpopulations, CD4+ and CD8+ T-cells **(A)**. Frequency of T-cells **(B)**, CD3+CD4+ **(C)**, and CD3+CD8+ **(D)** for healthy donors (HD, green circle), COVID-19 no invasive mechanical ventilation (No-IMV, purple triangle), and patients receiving IMV (orange square). FlowSOM tree analysis of T and B cells. For CD3+ T cells, clusters are shown as circles with star plots displaying the cluster median marker intensities. The background coloring represents the meta-clustering. Legends of the star plot and meta-clustering are shown on the right side **(E)**. Heatmap of the median marker intensities of the 18 markers across the 15 cell clusters obtained with the FlowSOM algorithm after the manual meta-cluster merging **(F)**. The color in the heatmap corresponds to the median of the arcsine-transformed marker expression (0–800 scaled) across cells from all samples. Blue represents a lower expression, while red represents a higher expression. The Uniform Manifold Approximation and Projection (UMAP) plot shows the 2D spatial distribution of 2.7 × 10^6^ cells from nine HD, nine No-IMV, and sixteen IMV. The colors in the UMAP plots represent the different populations obtained with the FlowSOM algorithm after the manual meta-cluster merging specified in the merge UMAP plot **(G)**. Analysis of cluster frequencies obtained by FlowSOM showed statistical differences between groups, with a population that decreased in COVID-19 patients **(H)**. Analysis of cluster frequencies by FlowSOM revealed statistical differences between groups, showing which populations increased in COVID-19 patients **(I)**. Data are represented as median and IQR values, and each dot represents an individual patient. The Kruskal-Wallis test was used to perform statistical comparisons; *p < 0.05, **p < 0.01, ***p < 0.001, ****p < 0.0001.

The frequency of total T-cells and CD4+ and CD8+ subsets was examined (**Figure 1A**). Compared to HD, T-cells were decreased in No-IMV (p= 0.0176) (**Figure 1B**), but CD4+ and CD8+ T-cell subsets were not modified (**Figures 1C, D**, respectively).

Using FlowSOM analysis and considering the expression of 18 parameters, fifteen T-cell subpopulations were identified by the FlowSOM tree analysis (**Figure 1E**). The intensity of the frequency of each subset is indicated in the heatmap (**Figure 1F**), and a percentual distribution of all populations is shown in **Supplementary Figure 3A**.

The two-dimensional distribution was visualized using UMAP, and notably, the T-cell subset distribution is different between COVID-19 status and HD (**Figures 1F, G**). Compared with HD, subsets 1–4 and 13 are decreased explicitly in IMV, whereas 5, 6, 10, and 12 are decreased in both No-IMV and IMV (**Figure 1H**). In contrast, 8 and 15 are increased in IMV compared with HD (**Figure 1I**). Other clusters, such as 7, 9, 11, and 14, did not show changes (**Supplementary Figure 3B**).

These data suggest that IMV COVID-19 patients exhibit a predominance of T-cell subsets associated with a increased inflammatory response, whereas No-IMV patients display a more regulated immune profile. The principal imbalance observed in IMV, but not in No-IMV, is characterized by an expansion of T cell subsets expressing activation and inflammation-related molecules (CD27, PD-L1, TNF-RI, IFN-γ RI [CD119], and CD95), alongside a reduction of subsets expressing regulatory receptors such as PD-1 and LAG-3 (9, 14, 15).

### 3.3 Decreased frequency of B-cells associated with suppressor function or migration capacity in IMV COVID-19 patients

**Figure 2:**
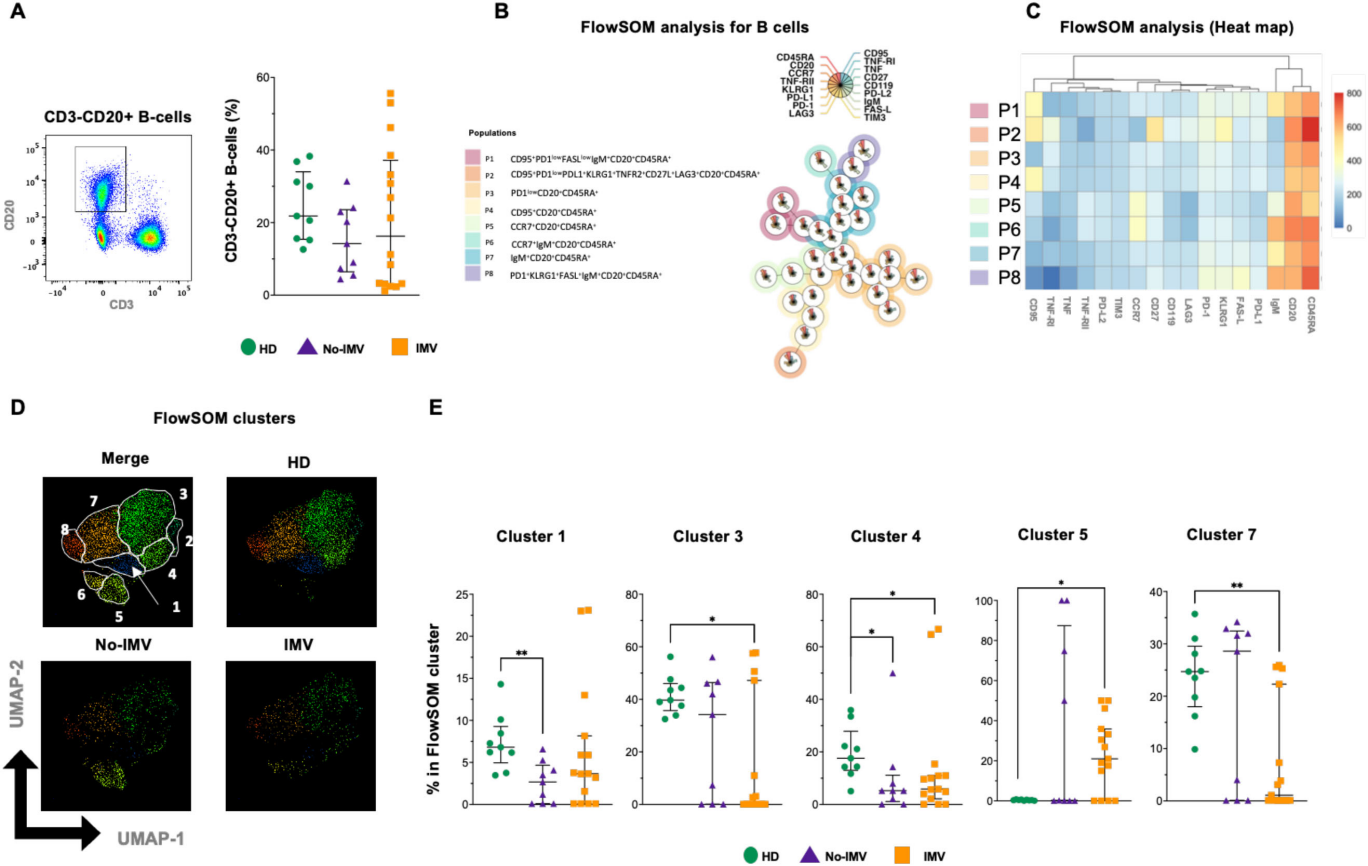
High-dimensional cytometry reveals differences in the distribution of B-cell subpopulations of COVID-19 patients. Representative dot plots showing manual gating analysis from CD3- cells to identify B-cells as CD20+IgM+/- **(A**, left), and B-cell frequency for healthy donors (HD, green circle), and COVID-19 patients: no invasive mechanical ventilation (No IMV, purple triangle), and patients received IMV (orange square) **(A**, right). FlowSOM tree analysis of B cells. For CD20+ B cells, clusters are shown as circles with star plots displaying the cluster median marker intensities. The background coloring represents the meta-clustering. Legends of the star plot and meta-clustering are shown on the right side **(B)**. Heatmap of the median marker intensities of the 17 markers across the 8 cell populations obtained with the FlowSOM algorithm after the manual metacluster merging **(C)**. The color in the heatmap indicates the median of the arcsine-transformed marker expression (0–800 scaled) across cells from all samples. Blue represents lower expression, while red represents higher expression. The Uniform Manifold Approximation and Projection (UMAP) plot shows the 2D spatial distribution of 6×10–^5^ cells from nine HD, nine No-IMV, and sixteen IMV. The colors in the UMAP plots represent the different populations obtained with the FlowSOM algorithm after the manual metacluster merging specified in the merge UMAP plot **(D)**. Analysis of cluster frequencies obtained from FlowSOM analysis showed statistical differences between groups, indicating that the population decreased or increased in COVID-19 patients **(E)**. Data are represented as median and IQR values, and each dot represents an individual patient. The Kruskal-Wallis test was used to perform statistical comparisons; *p < 0.05, **p < 0.01.

B-cells, the second strong arm of the adaptive immune response, were evaluated by flow cytometry (CD20+ cells). The total B-cell frequency was not different among the study groups (**Figure 2A**). In the FlowSOM analysis, considering the expression of 17 parameters, eight B-cell subpopulations were identified by the FlowSOM tree (**Figure 2B**); The intensity of the frequency of each subset is indicated in the heatmap (**Figure 2C**). A percentual distribution of all populations is shown in **Supplementary Figure 3C**.

Data showed that B-cell subpopulations have an imbalanced distribution (**Figure 2C**). The two-dimensional distribution was visualized using UMAP, and compared with HD, COVID-19 patients showed losses of B-cell subsets (**Figure 2D**). In particular, No-IMV showed decreased 1 and 4 subsets, and IMV decreased 3, 4, and 7 (**Figure 2E**). 2, 6, and 8 subsets did not change (**Supplementary Figure 3D**).

These results indicate that IMV patients exhibit a marked reduction in B-cell subsets associated with suppressive function (PD-1+, cluster 3), lymphoid homing (CCR7+, cluster 5), and IgM expression (cluster 7), a pattern not observed in No-IMV patients.

### 3.4 IMV COVID-19 has increased the expression of TNF pathway molecules in NKT subsets

Natural killer (NK) and natural killer T (NKT) cells play pivotal roles in modulating innate and adaptive immunity; they are lymphoid cells characterized by CD56 expression. While NK cells lack CD3 expression (CD3-), NKT cells are distinguished by CD3 expression (CD3+).

**Figure 3:**
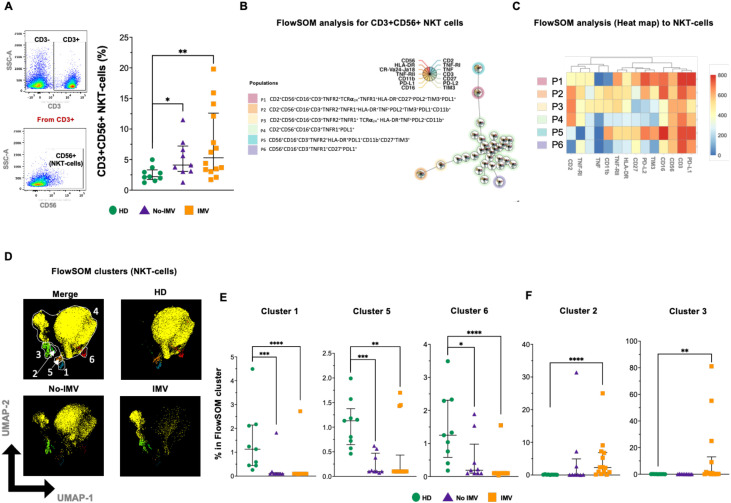
High-dimensional cytometry data reveal distinct subpopulation patterns of NKT cells in COVID-19 patients. Representative dot plots showing manual gating analysis of CD3 expression, then CD3+ events were plotted by CD56 expression to identify NKT cells **(A**, left). Analysis of cell frequencies for CD3+CD56+ NKT **(A**, right), for healthy donors (HD, green circle), and COVID-19 patients: no invasive mechanical ventilation (No-IMV, purple triangle), and patients received IMV (orange square). FlowSOM tree analysis of NKT cells. For CD3+CD56+ NKT cells, clusters are shown as circles with star plots displaying the cluster median marker intensities. The background coloring represents the meta-clustering. Legends of the star plot and meta-clustering are shown on the right side **(B)**. Heatmap of the median marker intensities of the 14 markers across the 6 cell populations obtained with the FlowSOM algorithm after the manual metacluster merging **(C)**. The color in the heatmap corresponds to the median of the arcsine-transformed marker expression (0–800 scaled) across cells from all samples. Blue represents a lower expression, while red represents a higher expression. The Uniform Manifold Approximation and Projection (UMAP) plot shows the 2D spatial distribution of 5.5 × 10^6^ cells from nine HD, nine No-IMV, and sixteen IMV. The color in the UMAP plots represents a different population obtained with the FlowSOM algorithm after manual metacluster merging, as specified in the merge UMAP plot **(D)**. Analysis of FlowSOM cluster frequencies revealed statistical differences between groups, with a decrease in the COVID-19 patient population **(E)**. Analysis of cluster frequencies using FlowSOM showed statistical differences between groups, with a population that increased in IMV patients **(F)**. Data are represented as median and IQR values, and each dot represents an individual patient. The Kruskal-Wallis test was used to perform statistical comparisons; *p < 0.05, **p < 0.01, ***p < 0.001, ****p < 0.0001.

NKT cells were identified by flow cytometry, and compared to HD, the total NKT frequency is increased in both No-IMV and IMV (**Figure 3A**). Considering 14 parameters, six NKT subpopulations were identified by the FlowSOM tree analysis (**Figure 3B**); a percentual distribution of all populations is shown in **Supplementary Figure 4A**.

The intensity of the frequency of each NKT subset is indicated in the heatmap (**Figure 3C**), and the two- dimensional distribution shows a loss of NKT subsets during COVID-19 (**Figure 3D**). Despite the two-dimensional distribution showing that IMV is more affected, the percentage graphics indicated that both No-IMV and IMV have decreased clusters 1, 5, and 6 (**Figure 3E**), while IMV has increased clusters 2 and 3 (**Figure 3F**), and cluster 4 did not show a change (**Supplementary Figure 4B**). These results suggest that No-IMV and IMV showed similar effects on some NKT subsets; however, IMV patients show an expansion of cluster 3, characterized by the expression of molecules associated with the TNF-TNFRs inflammation pathway. In contrast, this subset is not expanded in No-IMV patients, suggesting that TNF signaling may play a central role in the inflammatory signature observed in IMV.

### 3.5 IMV COVID-19 has increased NK cell subsets and higher expression of associated molecules, with a discrete expression of PD-1 pathway molecules

**Figure 4:**
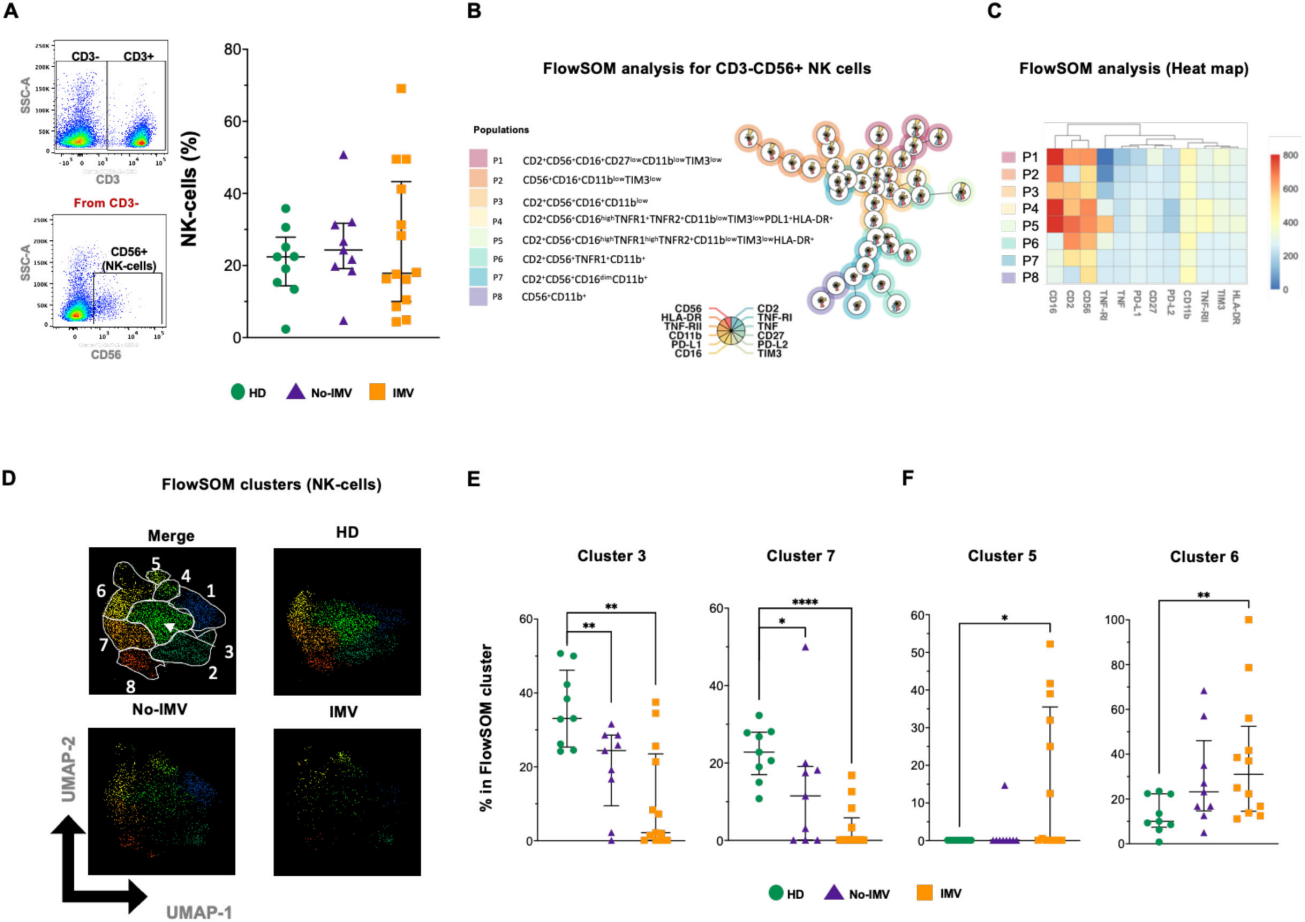
High-dimensional cytometry data reveal different subpopulation patterns in NK cells of COVID-19 patients. Representative dot plots showing manual gating analysis of CD3 expression, then CD3- events were plotted by CD56 expression to identify NK cells [**(A)** left]. The percentual distribution of all populations in each COVID-19 group and HD samples was plotted by color [**(A)** right] for healthy donors (HD, green circles), COVID-19patients without invasive mechanical ventilation (No-IMV, purple triangles), and COVID-19 patients receiving IMV (orange squares). FlowSOM tree analysis of NK cells. For CD3-CD56+ NK cells, clusters are shown as circles with star plots displaying the cluster median marker intensities. The background coloring represents the meta-clustering. Legends of the star plot and meta-clustering are shown on the right side **(B)**. Heatmap of the median marker intensities of the 12 markers across the 8 cell populations obtained with the FlowSOM algorithm after the manual metacluster merging **(C)**. The color in the heatmap corresponds to the median of the arcsine-transformed marker expression (0–800 scaled) across cells from all samples. Blue represents a lower expression, while red represents a higher expression. The A Uniform Manifold Approximation and Projection (UMAP) plot shows the 2D spatial distribution of 1×10–^5^ cells from nine HD, nine No-IMV, and sixteen IMV. The colors in the UMAP plots represent the different populations obtained with the FlowSOM algorithm after the manual metacluster merging specified in the merge UMAP plot **(D)**. Analysis of cluster frequencies obtained by FlowSOM showed statistical differences between groups, with a population that decreased in COVID-19patients **(E)**. Analysis of cluster frequencies using FlowSOM showed statistical differences between groups, with a population that increased in IMV patients **(F)**. Data are represented as median and IQR values, and each dot represents an individual patient. The Kruskal-Wallis test was used to perform statistical comparisons; *p < 0.05, **p < 0.01, ****p < 0.0001.

NK cells were also identified by flow cytometry, and we found that the frequency of total NK remains unaltered between groups (**Figure 4A**). Eight NK subsets were identified in the FlowSOM tree analysis (**Figure 4B**); The intensity of the frequency of each subset is indicated in the heatmap (**Figure 4C**). A percentual distribution of all populations is shown in **Supplementary Figure 4C**.

Although the two-dimensional distribution shows a loss of NK subsets during IMV COVID-19 (**Figure 4D**), the statistical analysis showed similar to the distribution of NKT subsets, clusters 3 and 7 were decreased in both No-IMV and IMV (**Figure 4E**). Conversely, subsets 5 and 6 were increased in the IMV (**Figure 4F**), whereas clusters 1, 2, 4, and 8 did not change (**Supplementary Figure 4D**). To note, NK subsets expanded specifically in IMV are characterized by higher expression of CD56, CD16, TNFRs, but relative lower expression of TIM-3 and PD-L1.This pattern further supports a potential role of TNF signaling in NK-cell-mediated inflammation in IMV patients.

### 3.6 IMV COVID-19 patients predominantly exhibit classical monocytes co-expressing TNFRs and IC

**Figure 5:**
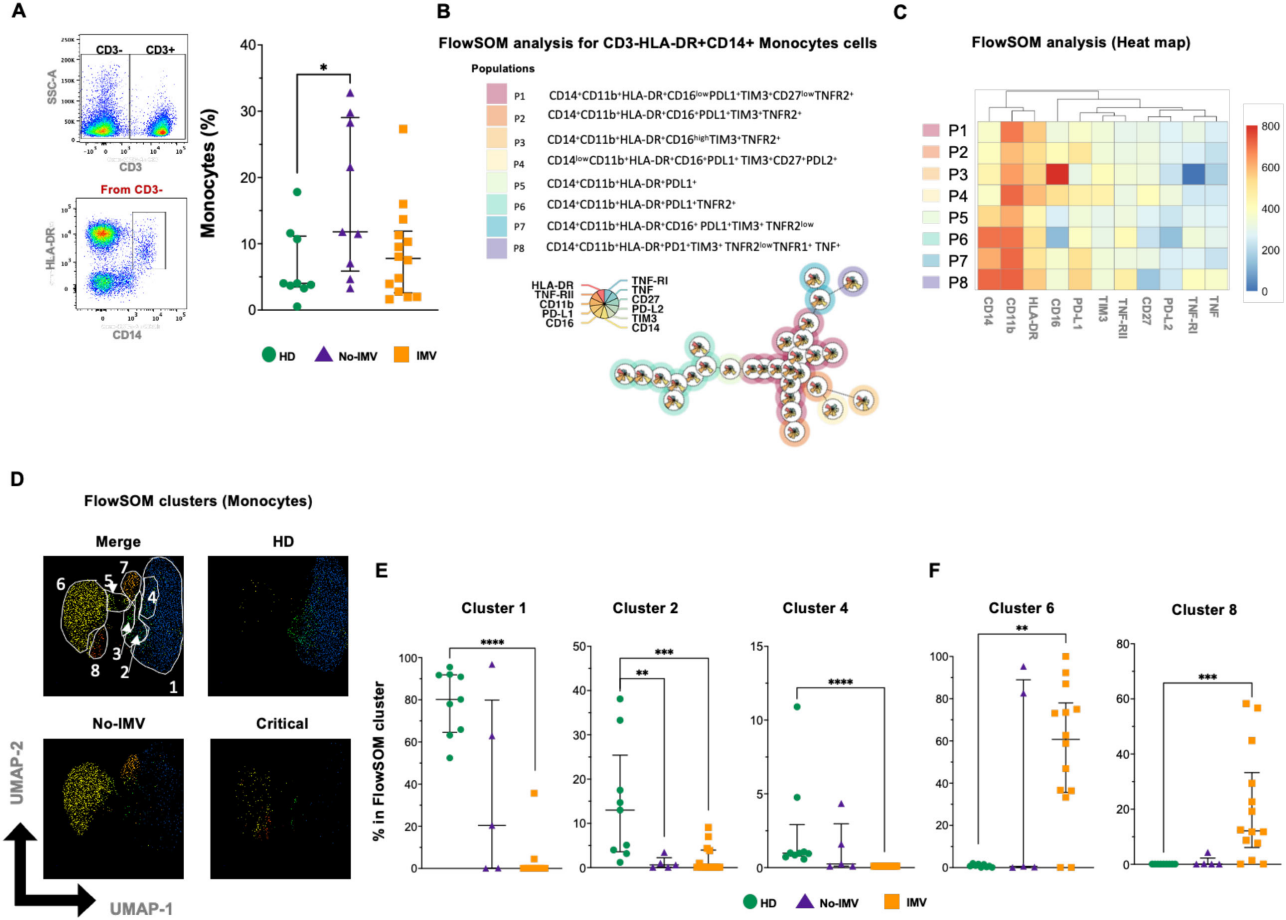
High-dimensional cytometry data reveal subpopulations with distinct patterns in monocytes from COVID-19 patients. Representative dot plots showing manual gating analysis of CD3 expression, then CD3- events were plotted by CD14 and HLA-DR expression to identify classical monocytes [**(A)** left]. Frequency of Classical monocytes (CD3-CD14+) for healthy donors (HD, green circle), and COVID-19 patients: no invasive mechanical ventilation (No IMV, purple triangle), and patients received IMV (orange square) [**(A)** right]. FlowSOM tree analysis of monocytes. For CD14+ monocytes, clusters are shown as circles with star plots displaying the cluster median marker intensities. The background coloring represents the meta-clustering. Legends of the star plot and meta-clustering are shown on the right side **(B)**. Heatmap of the median marker intensities of the 11 markers across the 8 cell populations obtained with the FlowSOM algorithm after the manual metacluster merging **(C)**. The color in the heatmap corresponds to the median of the arcsine-transformed marker expression (0–800 scaled) across cells from all samples. Blue represents a lower expression, while red represents a higher expression. A Uniform Manifold Approximation and Projection (UMAP) plot shows the 2D spatial distribution of 1×10–^5^ cells from nine HD, nine No IMV, and sixteen IMV. The colors in the UMAP plots represent the populations obtained with the FlowSOM algorithm after the manual metacluster merging specified in the merge UMAP plot **(D)**. Analysis of cluster frequencies obtained by FlowSOM revealed statistical differences between groups, showing that populations decreased in COVID-19 patients **(E)**. Analysis of cluster frequencies using FlowSOM showed statistical differences between groups, indicating populations that increased in COVID-19 patients **(F)**. Data are represented as median and IQR values. Each dot represents an individual patient. The Kruskal-Wallis test was used to perform statistical comparisons; * p < 0.05, **p < 0.01, ***p < 0.001, ****p < 0.0001.

From the gate CD3- cells, classical monocytes (CD14+HLA-DR+) were identified (**Figure 5A**). Considering 11 parameters, 8 classical monocyte subsets were identified by the FlowSOM tree analysis (**Figure 5B**). The intensity of the frequency of each subset is indicated in the heatmap (**Figure 5C**), and a percentual distribution of all populations is shown in **Supplementary Figure 5A**.

Compared to HD, No-IMV has an increased frequency of classical monocytes (p=0.0315) (**Figure 5A**). The two-dimensional distribution analysis shows the distribution of the monocyte subset (**Figures 5C, D**). Clusters 1 and 4 are decreased in IMV, while cluster 2 is decreased in both No-IMV and IMV (**Figure 5E**), whereas 6 and 8 subsets were increased in IMV (**Figure 5F**). Clusters 3, 5, and 7 did not show changes (**Supplementary Figure 5B**). These data showed that IMV and No-IMV display divergent monocyte subset profiles. IMV is characterized by a decrease in CD16+ monocyte (inflammatory profile) accompanied by an expansion of subsets co-expressing TNF-associated molecules and immune checkpoint regulatory molecules such as PD1, PD-L1, and TIM3.

### 3.7 COVID-19 Patients have decreased CD3+ monocyte co-expressing TNF-RII and IC

Studies have documented a non-classical subset of myeloid cells expressing CD3 that exhibit inflammatory properties. However, there is a significant gap in understanding the full scope of this monocyte subpopulation’s role. Because the functional relevance of this monocyte subpopulation remains poorly defined, we further evaluated this subset.

**Figure 6:**
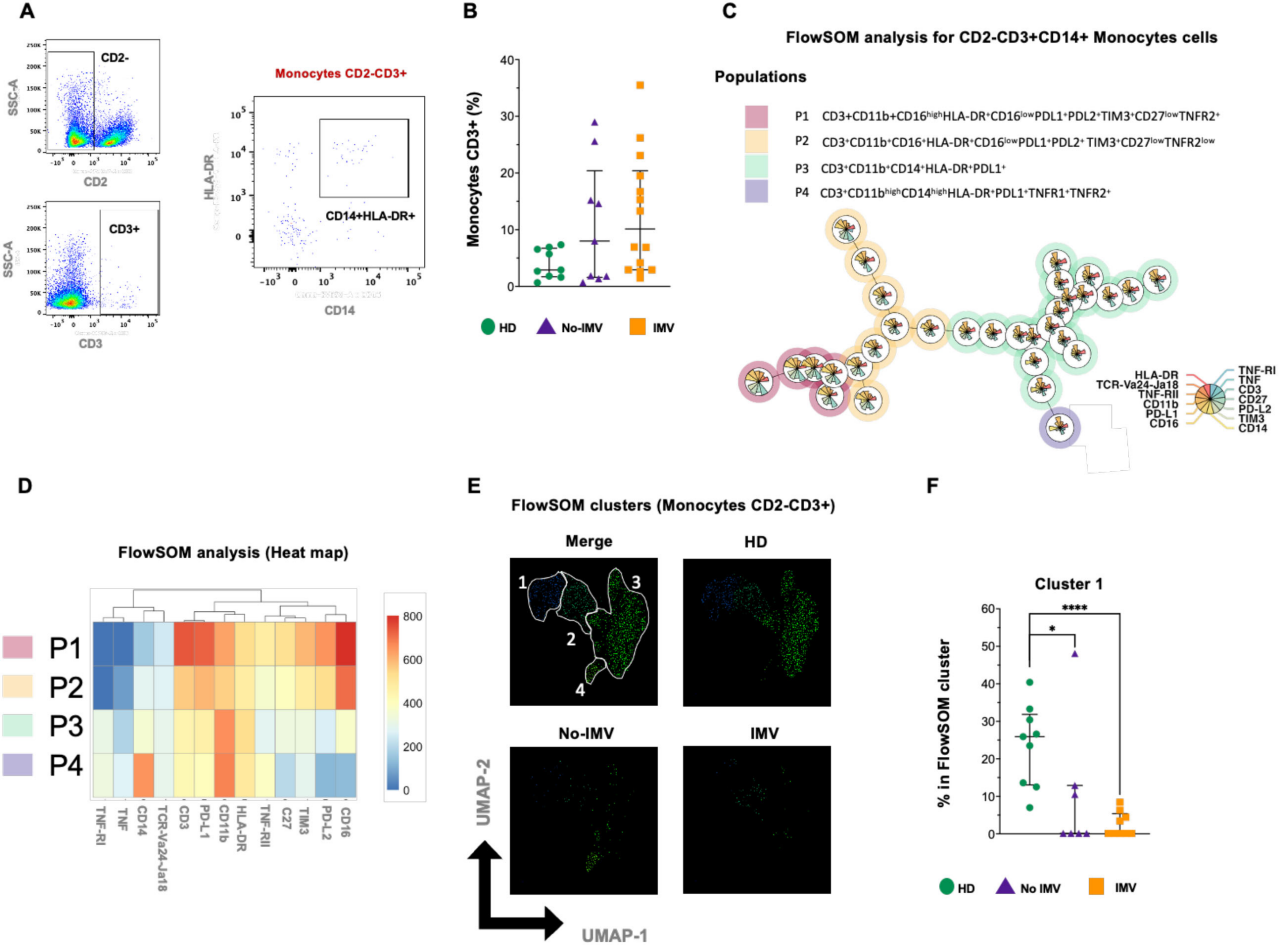
High-dimensional cytometry data reveal subpopulations with distinct patterns in CD3+ monocytes in COVID-19 patients. Representative dot plots showing manual gating analysis from CD2- cells to identify CD3+ cells, then CD3+ monocytes were identified as CD2-CD3+CD14+HLA-DR+ **(A)**. Frequency of non-classical CD3+ monocytes for healthy donors (HD, green circle) and COVID-19 patients: no invasive mechanical ventilation (No IMV, purple triangle), and patients who received IMV (orange square) **(B)**. FlowSOM tree analysis of monocytes. For CD14+ monocytes, clusters are shown as circles with star plots displaying the cluster median marker intensities. The background coloring represents the meta-clustering. Legends of the star plot and meta-clustering are shown on the right side **(C)**. Heatmap of the median marker intensities of the 13 markers across the 4 cell populations obtained with the FlowSOM algorithm after the manual metacluster merging **(D)**. The color in the heatmap corresponds to the median of the arcsine-transformed marker expression (0–800 scaled) across cells from all samples. Blue represents a lower expression, while red represents a higher expression. The Uniform Manifold Approximation and Projection (UMAP) plot shows the 2D spatial distribution of 2.6 × 10^3^ cells from nine HD, nine No IMV, and sixteen IMV. The colors in the UMAP plots represent the different populations obtained with the FlowSOM algorithm after the manual metacluster merging specified in the merge UMAP plot **(E)**. Analysis of cluster frequencies obtained by FlowSOM revealed statistical differences between groups, showing that populations decreased in COVID-19 patients **(F)**. Data are represented as median and IQR values, and each dot represents an individual patient. The Kruskal-Wallis test was used to perform statistical comparisons; *p < 0.05, ****p < 0.0001.

Excluding CD2+ cells, the phenotype CD3+CD14+HLA-DR+ was identified by flow cytometry (**Figure 6A**), and the data showed that the frequency of non-classical CD3+ monocytes was not modified across the different COVID-19 groups (**Figure 6B**). In the FlowSOM tree analysis with 13 parameters, four non-classical monocyte subpopulations were identified (**Figure 6C**), and the percentual distribution of all populations is shown in **Supplementary Figure 5C**.

The intensity of the frequency of each subset is indicated in the heatmap, and the two-dimensional distribution analysis shows a loss of CD3+ monocyte subsets, mainly in IMV (**Figures 6D, E**). Cluster 1 is altered in both COVID-19 groups, although the effect is more pronounced in IMV patients. In contrast, No-IMV patients retain a profile closer to baseline, and clusters 2–4 remain largely unaffected in both groups (**Figure 3F** and **Supplementary Figures S5D**). Notably, the affected cluster is characterized by co-expression of TNFR-II and immune checkpoint molecules (PD-L1, PD-L2, and TIM-3), whereas in classical monocytes, a phenotypically comparable cluster is increased.

### 3.8 T-cells from no-IMV exhibit impaired activation of cytotoxic cells in response to polyclonal stimuli

We investigated T cell activation capacity to determine whether the imbalance in cell subsets during COVID-19 led to a weak immune response.

**Figure 7:**
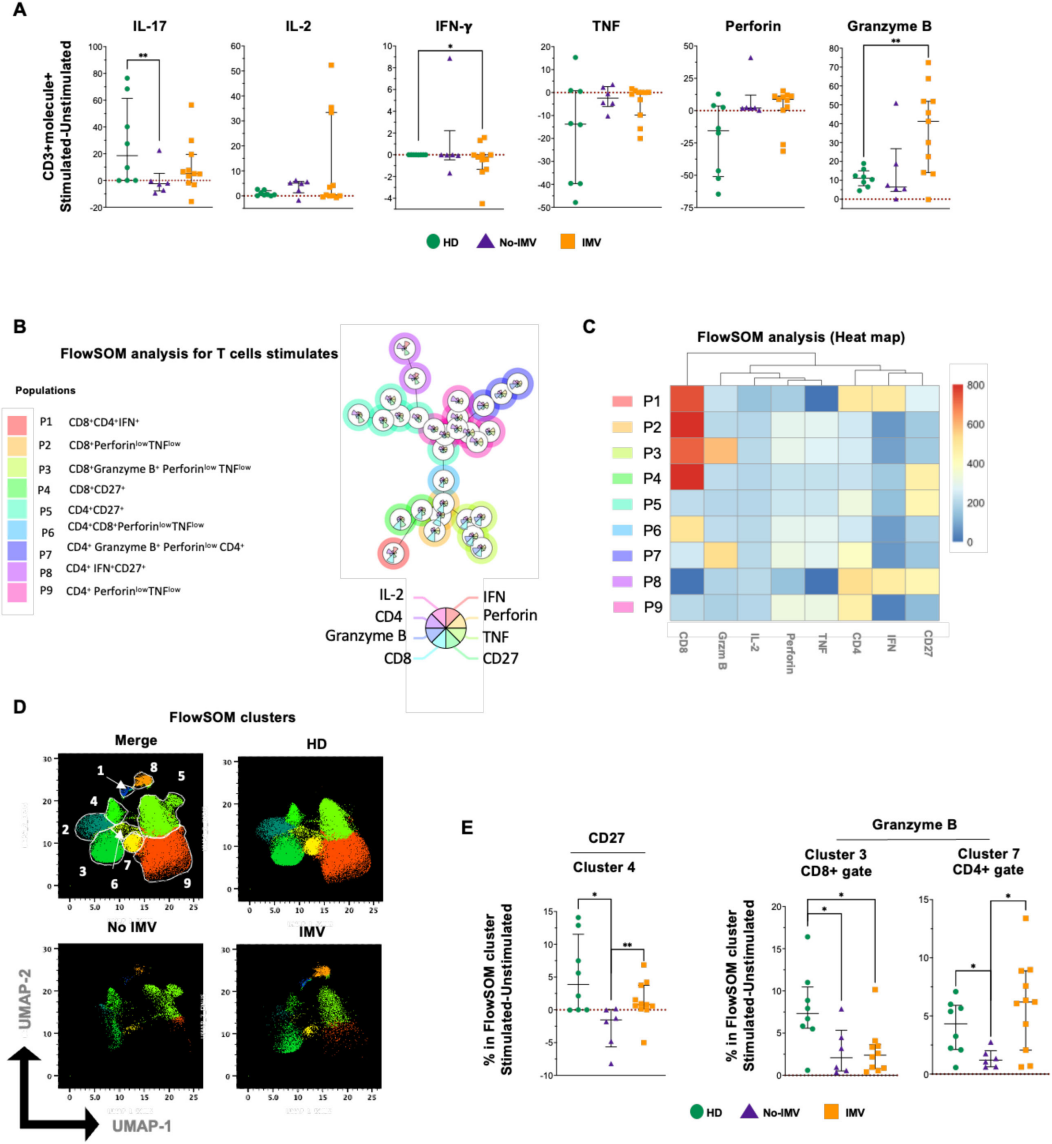
High-dimensional cytometry data reveal distinct T-cell activation patterns in COVID-19 patients. Cells of eight healthy donors (HD, green circle), six COVID-19 patients who did not receive invasive mechanical ventilation (No-IMV, purple triangle), and eleven who received IMV (orange square) were cultured with PMA/Iono for 6 hours. Soluble molecules were measured in the culture supernatant using a LegendPlex kit. Graphics are presented as percentages, with levels in the unstimulated condition normalized to zero (dotted red line) **(A)**. Cells were recovered at the end of culture and prepared for flow cytometry. FlowSOM tree analysis of T cells. For CD3+ CD4+ and CD8+ T cells, clusters are shown as circles with star plots displaying the cluster median marker intensities. The background coloring represents the meta-clustering. Legends of the star plot and meta-clustering are shown on the right side **(B)**. A heatmap of the median marker intensities for the 8 markers and 9 cell populations was generated using the FlowSOM algorithm after manual metacluster merging. The color in the heatmap indicates the median of the arcsine-transformed marker expression across all cells, with blue representing lower expression and red representing higher expression **(C)**. The Uniform Manifold Approximation and Projection (UMAP) plot shows the 2D spatial distribution of cells from HD, No-IMV, and IMV. The color in the UMAP plots represents a different population obtained with the FlowSOM algorithm after manual metacluster merging, as specified in the merge UMAP plot **(D)**. Analysis of cluster frequencies obtained by FlowSOM analysis showed statistical differences (stimulated-unstimulated) between groups, indicating the affected clusters **(E)**. Data are represented as median and IQR values, and each dot represents an individual patient. The Kruskal-Wallis test was used to perform statistical comparisons; *p < 0.05, **p < 0.01.

Soluble levels of cytokines and cytotoxic factors were evaluated after a polyclonal stimulus. Considering its unstimulated condition as zero (dotted red line), cells from COVID-19 patients show a reduced capacity to deliver some proinflammatory cytokines compared to HD. No-IMV cells exhibited a reduced capacity to produce IL-17, whereas IMV showed impaired IFN-γ production, suggesting that distinct inflammatory pathways are disrupted in IMV and No-IMV. IL-2 and TNF remained unchanged in both groups (**Figure 7A**). Moreover, IMV and No-IMV differed in cytotoxic mediators production, as IMV cells secreted higher levels of granzyme B, while perforin expression remained unchanged (**Figure 7A**).

To further analyze, some of these molecules were evaluated at the intracellular level in CD4+ and CD8+ T-cell subsets. Considering eight parameters, the FlowSOM tree analysis identified nine T-cell subpopulations (**Figure 7B**), and the intensity of the frequency of each subset is indicated in the heatmap (**Figure 7C**). The distribution of cell subsets before (left of the black line) and after stimulation (right of the black line) is shown in S6A. The two-dimensional distribution further indicates that cell subsets from COVID-19 patients exhibit reduced responsiveness to activation (**Figure 7D**).

The percentage of subsets was normalized to the basal percentage, set to zero (dotted red line) (**Figure 7E** and **Supplementary Figure S6B**). Thus, in response to polyclonal stimuli, No-IMV loss reduced cluster 4, whereas IMV generated clusters similar to HD (**Figure 7E**). Additionally, No-IMV and IMV have affected the presence of CD8+ T cells co-expressing Granzyme B and TNF (cluster 3). However, IMV induce the presence of CD4+ T cells with phenotype reminiscent of the subset called CD4+ cytotoxic (cluster 7, **Figure 7E**), which are reported to arise as a compensatory mechanism for defective cytotoxic, additionally, the cluster 9 (CD4+ perforin+) is maintained in No-IMV and IMV as response to the stimuli but it is loss in HD (**Supplementary Figure 6B**).

## 4 Discussion

In this study, we analyzed a broad range of immune cell subsets, including T cells, B cells, NK cells, NKT cells, and monocytes, in COVID-19 patients, categorizing them based on the severity of the disease according to the use or non-use of IMV. We also assessed their activation capacity in response to polyclonal stimuli. Our study used a high-dimensional panel, enabling us to characterize the distribution of diverse cell subsets, complemented by UMAP analysis to visualize their two-dimensional spatial distribution. All evaluated subsets in this study should not be interpreted as isolated phenomena, the immune responses operate through highly interconnected cellular networks in which the functional state of one population influences others. This interdependence is particularly relevant in diseases characterized by hyperinflammation and dysregulated immunotypes, such as COVID-19, where immune imbalance constitutes a central component of pathophysiology. Thus, defining the immune landscape during SARS-CoV-2 infection may provide critical insights to identify potential targets for the development of coordinated therapeutic interventions.

We identified that IMV and No-IMV exhibit distinct immune cell subset profiles. According to disease severity and the requirement for invasive mechanical ventilation, patients demonstrate specific gains and losses of immune subsets. These coordinated alterations likely contribute to the establishment of distinct immune landscapes, which may ultimately influence clinical outcomes. In consonance to previous reports, perturbations in T-cell subsets have been associated with disease severity, a phenomenon linked to increased apoptosis. More recently, it has been suggested that severe COVID-19 leads to a decline in TCF1+ progenitor T cells, impairing their capacity for proliferation and self-renewal (16–18). The data showed that IMV patients primarily lose T-cell subsets expressing IC, such as PD-1 and LAG-3 (clusters 1-6, 10, 12, 13, **Figure 1**). Conversely, IMV patients exhibit a predominance of T cells co-expressing molecules involved in the TNF/TNFR-mediated inflammatory pathway and in cell death, such as CD95-L (cluster 15).

In viral infections, the LAG-3 and PD-1 pathways are essential for regulating lymphocyte activation (19, 20). A recent study suggested that CD4+ memory T cells expressing LAG-3 constitute a risk factor for COVID-19 infection (21). Similarly, PD-1 and its ligands have been proposed as prognostic markers and potential therapeutic targets in severe COVID-19 cases (22). Moreover, increased PD-L2 expression and an altered CD4/CD8 ratio have been associated with persistent post-COVID-19 lung lesions (13).

Our data demonstrate that T-cell subsets are markedly affected by COVID-19 severity. In particular, the increased frequency of TNF/TNFR-positive T cells likely contributes to the proinflammatory microenvironment observed in severe disease. This finding is consistent with previous reports indicating that COVID-19 severity is associated with a higher prevalence of TNFR1+ cells, which can interact with TNF, promoting the activation of cell death pathways (6). In parallel, the increased frequency of T cells co-expressing CD95-L may be linked to the induction of PANoptosis, a form of inflammatory cell death characterized by the simultaneous activation of multiple programmed cell death pathways. This mechanism may perpetuate inflammation and lead to an uncontrolled, deleterious inflammatory state, as previously described in severe infectious (23).

CD27 is a critical costimulatory receptor that promotes T-cell activation, survival, and long-term immune protection by recruiting intracellular signaling molecules involved in canonical and noncanonical pathways, including MAPK signaling. CD27-driven co-stimulation has been proposed as a key mechanism for generating memory T-cell clones with enhanced functional potential, particularly within the CD8+ T-cell compartment (24, 25). In this context, our data show that IMV-COVID-19 patients exhibit a marked deficiency of CD27-expressing T cells (clusters 1–3, 4 and 13, **Figure 1**), predominantly affecting CD8+ T cells.

Notably, in response to the activation stimulus, IMV and No-IMV patients displayed a differential redistribution of CD27+ T-cell subsets (**Figure 7** and **Supplementary Figure S6**). Only IMV showed an increased frequency of CD4+CD27+ and CD8+CD27+ T cells (clusters 5 and 4, respectively), whereas the frequency of IFN-γ–secreting CD4+CD27+ T cells was comparable between groups (cluster 8). Collectively, these findings suggest that both IMV and No-IMV patients retain the capacity to sustain an IFN-γ–dependent inflammatory response. Nevertheless, only IMV patients exhibited an expansion of additional CD27+ subsets upon activation, despite their reduced baseline frequencies. This pattern may reflect a compensatory attempt to enhance cytotoxic activation in the context of severe disease. However, this response appears insufficient, as CD8+ T cells expressing lytic molecules remain functionally impaired. In contrast, No-IMV patients may not require further upregulation of CD27 expression within these subsets, possibly reflecting a more balanced immune activation profile.

In this regard, despite the reduced frequency of CD8+Granzyme B+ T cells (cluster 3, **Figure 7**), soluble Granzyme B levels were increased following activation. This apparent discrepancy can be explained by the significant expansion of a CD4+ T-cell subset expressing Granzyme B (cluster 7, **Figure 7**). Then, these findings suggest that CD8+ T cells in IMV-COVID-19 patients may have an impaired capacity to respond to activation stimuli, thereby promoting the appearance of cytotoxic CD4+ T cells as a compensatory mechanism. The accumulation of these cells has been reported in diverse contexts, including chronic infections and normal aging (26). Although the precise origin and differentiation pathways of these cells remain incompletely understood, evidence from infectious disease models indicates that CD8+ T-cell exhaustion can favor the development of cytotoxic CD4+ T cells, potentially contributing to disease control (26, 27). Supporting this notion, single-cell transcriptomic analyses have identified cytotoxic follicular CD4+ T cells responding to SARS-CoV-2 infection, which, due to their phenotype and localization, may be associated with impaired humoral responses (28).

Previous reports have indicated an imbalance in B-cell subsets in COVID-19, associated with a lack of follicular T cells (29). Similarly, we observed a reduction in a CCR7-expressing B-cell subset, a receptor essential for lymphocyte migration to secondary lymphoid organs. This finding raises the possibility that severe COVID-19 may have long-term consequences on B-cell trafficking and immune organization. Indeed, persistent B-cell lymphopenia has been documented in critically ill COVID-19 patients up to 50 days after symptom onset (30). Recently, it has been shown that impaired B-cell function and an expansion of unswitched B cells in patients with residual lung abnormalities up to 12 months after critical disease (31). Regarding IC expression, we did not detect strong expression in B cells, and although our findings show weak PD-1 expression, ligand expression was not detectable. This observation is in line with previous studies reporting PD-1 expression across multiple B-cell subsets, ranging from naïve to memory populations, suggesting a role of PD-1 in B-cell dysregulation during severe COVID-19 (32).

It is well established that the generation of optimal humoral immunity requires coordinated interactions between B and T cells. Thus, the T-cell profiles identified in this study may have direct implications for quality of long-term humoral responses. Previous studies have demonstrated that acute COVID-19 patients exhibit reduced frequencies of memory B cells and follicular helper T cells, which negatively impact the development of durable humoral immunity (33). In this context, recent reports suggest that alterations in CD27-expressing subsets may predict the development of long COVID (34). Furthermore, even in vaccinated individuals, evidence indicates that protection against breakthrough infections requires both robust T-cell responses and high titers of neutralizing antibodies (35). Together, these observations reinforce the concept that immune alterations during the SARS-CoV-2 infection should be understood within the framework of coordinated immune networks rather than isolated cellular alterations, as disruption in one compartment may compromise systemic antiviral immunity.

NK and NKT cells are low-frequency homogeneous cell groups. As with T and B cells, IMV patients exhibit a significant loss of NK and NKT cell subsets. This finding is consistent with previous reports demonstrating that severe SARS-CoV-2 infection leads to abnormal NK and NKT cell features. Despite their decreased numbers, these cells exhibit aberrant expression of natural cytotoxicity receptors (36). For the first time, our study identified that both NK and NKT cells expressing TNF/TNFRs are more abundant in IMV-COVID-19 patients. Given the known role of this pathway, it is likely that cell death is an active mechanism affecting these subsets in critical COVID-19 cases.

Finally, the presence of monocyte subsets was severely modified. Although the total monocyte frequency did not differ significantly between groups, certain subsets were almost absent. This aspect requires further investigation, as existing reports are controversial. One study indicated that in severe COVID-19, decreased monocyte-associated HLA-DR expression is linked to a higher risk of death (37). Similarly, another study found that patients who died had significantly lower HLA-DR expression on classical monocytes (CD14+CD16-) (38), suggesting that early HLA-DR reduction in monocytes could serve as a predictor of mortality. In contrast, a 2022 study reported an enrichment of classical monocytes (CD16-) in severe COVID-19 patients, with these cells exhibiting abnormal mitochondrial superoxide levels and lipid peroxidation (38). Additionally, it is important to note that the affected classic monocyte subsets do not express immune checkpoints, such as PD-L1, PD-L2, and TIM-3. In contrast, the affected CD3+ monocyte subset expresses these markers, suggesting that classic monocytes retain regulatory functions, whereas CD3+ monocytes do not.

In this way, monocytes should not be considered as isolated effectors but as integral components of this immune network, they establish dynamic interactions with lymphocyte populations and actively shape inflammatory responses. A reduced HLA-DR expression on monocytes has been associated with increased disease severity and elevated inflammatory markers in COVID-19 patients, authors proposed that the monocyte-to-lymphocyte ratio can be used as an early indicator of bacterial sepsis in individuals with severe COVID-19 (39). In addition, decreased HLA-DR expression on monocytes combined with low CD4+ T-cell counts has been associated with increased mortality risk in severe COVID-19 patients following dexamethasone treatment, authors support the need to integrate comprehensive immune monitoring into the clinical management of critically ill patients in order to guide more precise immunomodulatory strategies (40). Taken together, these observations emphasize that viral pathogenesis and disease progression are shaped by coordinated, multi-compartment immune interactions rather than by alterations in single cell types.

In summary, our results demonstrate that IMV and No-IMV patients display clearly distinct immune landscapes. IMV patients are characterized by exhibiting a profound imbalance in the immune cell network, affecting CD8+ T cells and leading to the emergence of non-conventional cytotoxic CD4+ T cells to compensate for the deficiency in cytotoxic function. In contrast, No-IMV maintains a more stable distribution of immune cells. The most significant alterations were observed in T cells, with a predominance of TNF/TNFR-expressing T cells and a significant downregulation of IC-expressing subsets, including PD-1, LAG-3, and KLRG1. Regarding B cells, we observed a decrease in the IgM+ and CCR7+ subsets, suggesting impaired migration to secondary lymphoid organs. Finally, IMV patients displayed a predominance of TNF/TNFRs-expressing NK and NKT cells. Collectively, these findings support the notion that severe illness is associated with a disruption of immune cell homeostasis. Although the design in this study does not allow us to establish definitive causality, these data suggest a bidirectional relationship, where severe disease may initially drive immune dysregulation, which in turn perpetuates immune imbalance and further amplifies tissue damage. Then, considering this model, immune dysfunction is not merely a consequence of severe illness but becomes an active contributor to disease progression, establishing a self-reinforcing pathological loop.

## Data Availability

The original contributions presented in the study are included in the article/**Supplementary Material.** Further inquiries can be directed to the corresponding author.
