# Supplementary material for: Imbalanced immune cell network and suboptimal cell activation: signatures associated with disease severity in vaccine-naïve COVID-19 patients
Source: Front Immunol. 2026 Mar 10;17:1794103. doi: 10.3389/fimmu.2026.1794103 (PMC13008623; doi:10.3389/fimmu.2026.1794103)
Supplement: Supplementary file 1 [file DataSheet1.pdf]

Supplementary Material

1 Supplementary Figures and Tables

1.1 Supplementary Tables

ANTIBODY	FLUOROCHROME	BRAND	REFERENCE
ANTI-CD27	Brilliant Ultraviolet 395	BD Bioscience	563815
ANTI-CCR7	PE	BioLegend	353204
ANTI-CD119 (IFN-GRI)	Brilliant Ultraviolet 496	BD Bioscience	750409
ANTI-CD11B	BV785	BioLegend	301346
ANTI-CD14	Brilliant Violet 510	BioLegend	301842
ANTI-CD16	Brilliant Violet 650	BioLegend	302042
ANTI-CD19	Brilliant Violet 615	BD Bioscience	564456
ANTI-CD2	APCH7/APCCY7	BioLegend	309238
ANTI-CD20	PE-Cy5	BioLegend	302308
ANTI-CD3	Brilliant Blue 700	BD Bioscience	566575
ANTI-CD4	Brilliant Violet 510	BioLegend	357420
ANTI-CD45RA	PE-Cy7	BioLegend	304126
ANTI-CD56	PE-Cy7	BioLegend	318318
ANTI-CD8	Brilliant Ultraviolet 563	BD Bioscience	612914
ANTI-CTLA-4	KIRAVIA Blue 520	BioLegend	349938
ANTI-FAS (CD95)	APC/Cy7	BioLegend	305636
ANTI-FAS-L	Brilliant Ultraviolet 737	BD Bioscience	748972
ANTI-GRANZYME B	Brilliant Violet 421	BioLegend	396414
ANTI-HLA-DR	PE-Cy5	BioLegend	307608
ANTI-IFN-G	APC/Cy7	BioLegend	502530
ANTI-IGM	Brilliant Ultraviolet 661	BD Bioscience	750365
ANTI-IL-2	FITC	BioLegend	500304
ANTI-KLRG1	Brilliant Violet 785	BioLegend	138429
ANTI-LAG-3	Brilliant Violet 605	BioLegend	369324
ANTI-PD-1	Brilliant Violet 650	BioLegend	367430
ANTI-PD-L1	Brilliant Violet 711	BioLegend	329722
ANTI-PD-L2	Brilliant Ultraviolet 615	BD Bioscience	751538
ANTI-PERFORIN	APC	BioLegend	353312
ANTI-TCR VA24-JA18	PE	BioLegend	342904
ANTI-TIM-3	Brilliant Violet 421	BioLegend	345008
ANTI-TNF	Alexa Fluor 700	BioLegend	502928
ANTI-TNF-RI	APC	BioLegend	369906
ANTI-TNF-RII	PE/Dazzle 594	BioLegend	358414

Table S1. Antibodies used in the study.

1.2 Supplementary Figures

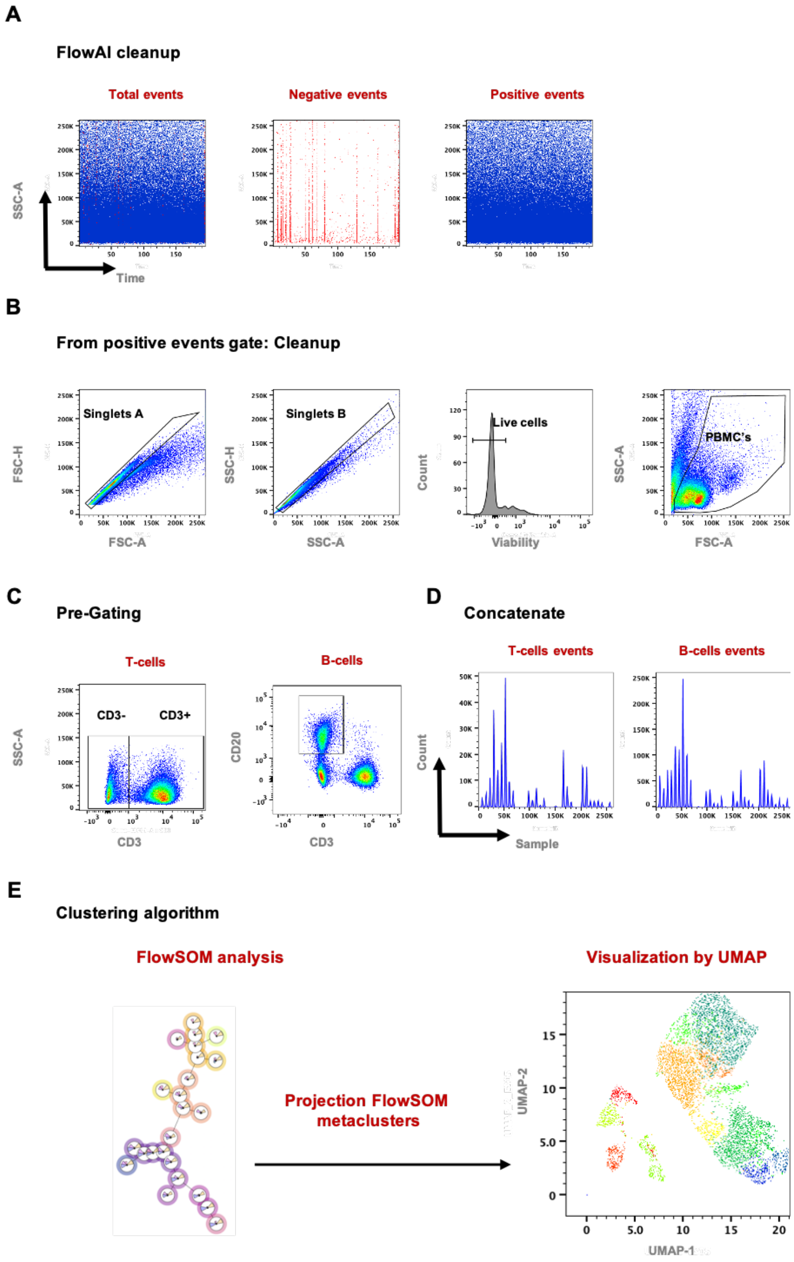

Figure S1. Representative data preprocessing was performed before the computational analysis of T and B cells using the clustering algorithm. The FlowAI analysis was conducted as a quality control step to detect and remove anomalies from FCS data, evaluating flow rate, signal acquisition, and dynamic range in each sample acquired before analysis (A). Representative dot plots showing manual gating analysis of positive events gate; first, from singlet cells were selected, followed by single events identified by forward (FSC-A vs. FSC-H) and side scatter (SSC-H vs. SSC-A) dot plot. Then, cell viability was evaluated, and mononuclear cells were selected by forward scatter (FSC) and side scatter (SSC) dot plots (B). For each .fsc file, a pre-gating was performed based on CD3 expression to select T cells. From CD3- events, CD20 and IgM expression are used to select B cells, ensuring that downstream analysis includes only clean T cell or B cell data. (C). All samples were concatenated into T and B cell groups for analysis of each group (D). Each population was studied using a FlowSOM algorithm after manual metacluster merging, and the metaclusters were projected onto a Uniform Manifold Approximation and Projection (UMAP) plot (E).

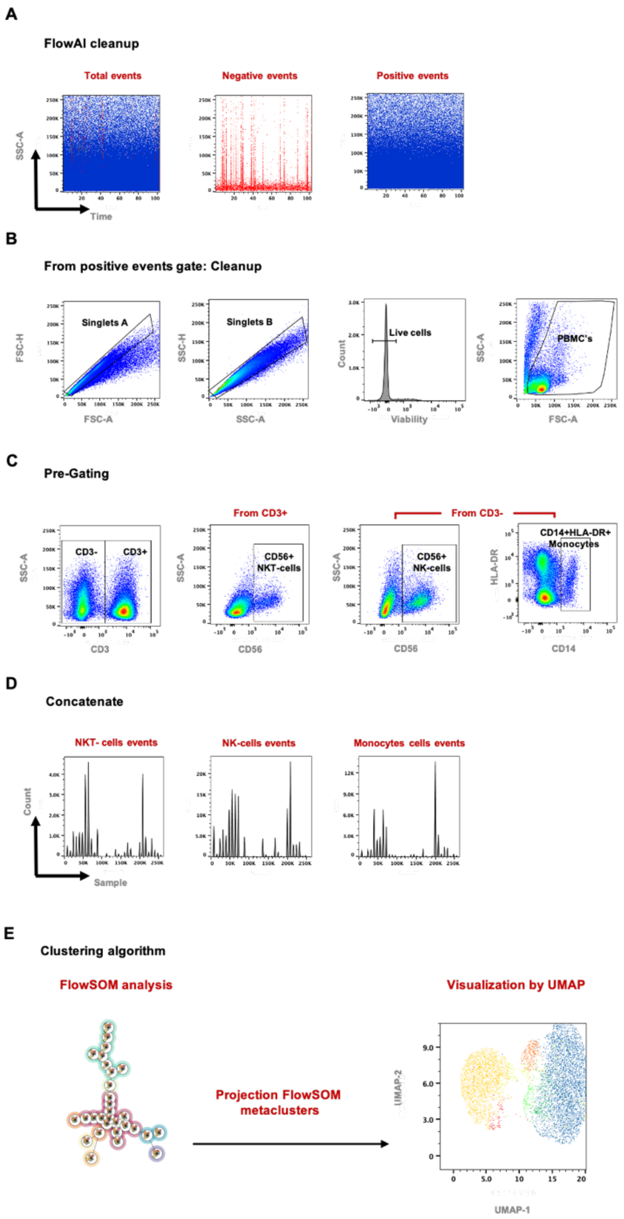

Figure S2. Representative data preprocessing was performed before the computational analysis of NKT, NK, and monocyte cells using the clustering algorithm. The FlowAI analysis was conducted as a quality control step to detect and remove anomalies from FCS data, evaluating flow rate, signal acquisition, and dynamic range in each sample acquired before analysis (**A**). Representative dot plots showing manual gating analysis of positive events gate; first, from singlet cells were selected, followed by single events identified by forward (FSC-A vs. FSC-H) and side scatter (SSC-H vs. SSC-A) dot plot. Then, cell viability was evaluated, and mononuclear cells were selected by forward scatter (FSC) and side scatter (SSC) dot plots (B). For each .fsc file, a pre-gating was performed based on CD3 expression. Among CD3- events, CD56+ cells were identified as natural killer cells (NK cells), and the co-expression of CD14 and HLA-DR was considered indicative of total monocytes (CD14+HLA-DR+). Among CD3+ events, CD56+ cells were identified as natural killer T cells (NKT). Each population was identified in all samples to ensure that only clean NKT, NK, and monocyte cell data were included in the downstream analysis. (C). All samples were concatenated into NKT, NK, and monocyte groups for analysis within each group (D). Each population was studied by first creating a FlowSOM algorithm after manual metacluster merging, and the metaclusters were projected onto a Uniform Manifold Approximation and Projection (UMAP) plot (E).

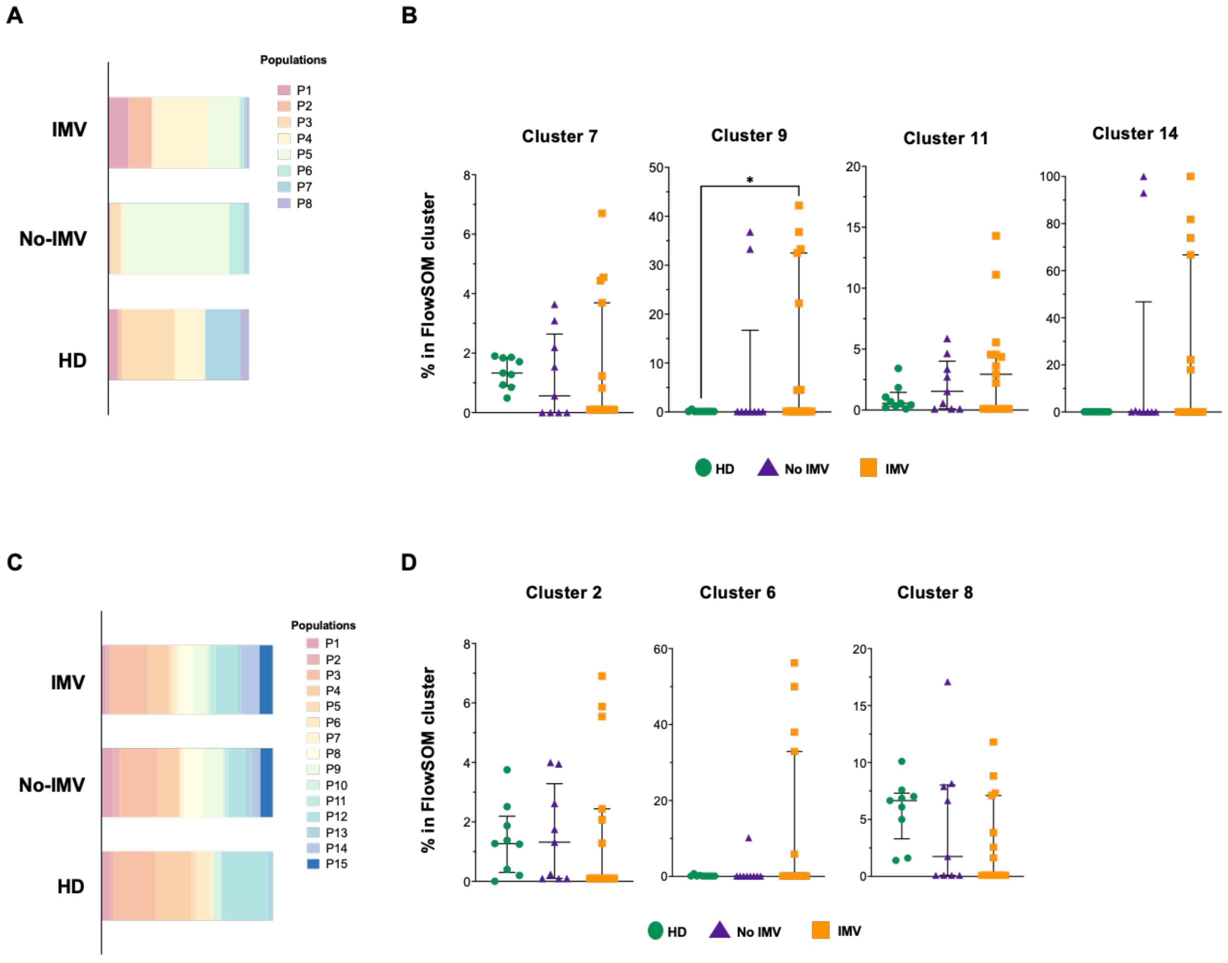

Figure S3. For the CD3+ T cells, the percentual distribution of all populations in each COVID-19 group and HD samples was plotted by color (**A**). Analysis of cluster frequencies without statistical differences between groups of CD3+ T cells (B). For the CD20+ B cells, the percentual distribution of all populations in each COVID-19 group and HD samples was plotted by color (C). Analysis of cluster frequencies without statistical differences between groups to CD20+IgM+/- B cells (D). Data are represented as median and IQR values, and each dot represents an individual patient. The Kruskal-Wallis test performed statistical comparisons. A control group was identified as HD, a healthy donor (green circle). COVID-19 patients were divided into No IMV (purple triangle), IMV: severe (orange square), and IMV: critical (red hexagon).

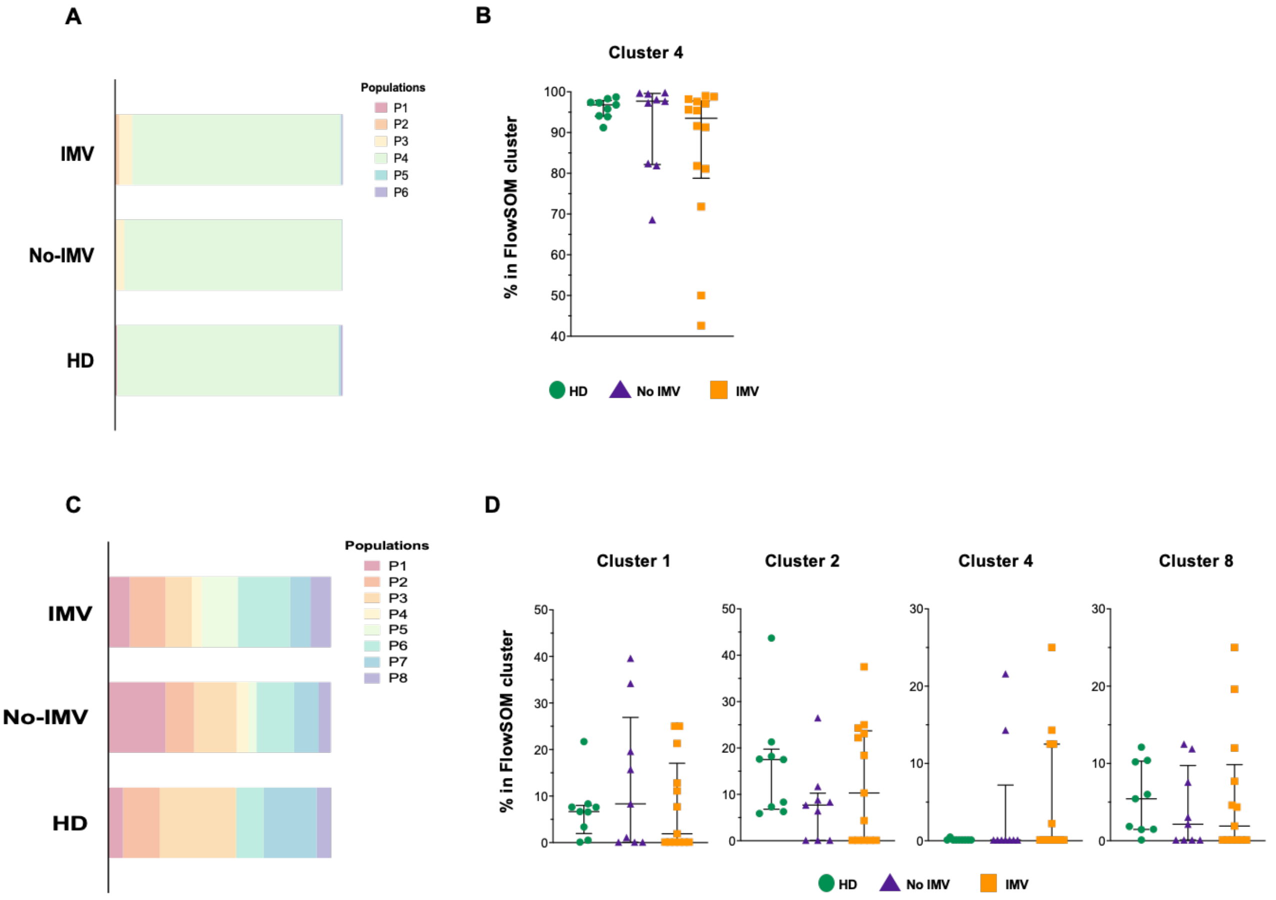

Figure S4. FlowSOM tree analysis of NKT and NK cells. For CD3+CD56+ NKT cells, the percentual distribution of all populations in each COVID-19 group and HD samples was plotted by color (A). Analysis of cluster frequencies without statistical differences between groups to CD3+CD56+ NKT cells (B). For CD3-CD56+ NK cells, the percentual distribution of all populations in each COVID-19 group and HD samples was plotted by color (C). Analysis of cluster frequencies without statistical differences between groups to CD3-CD56+ NK cells (D).

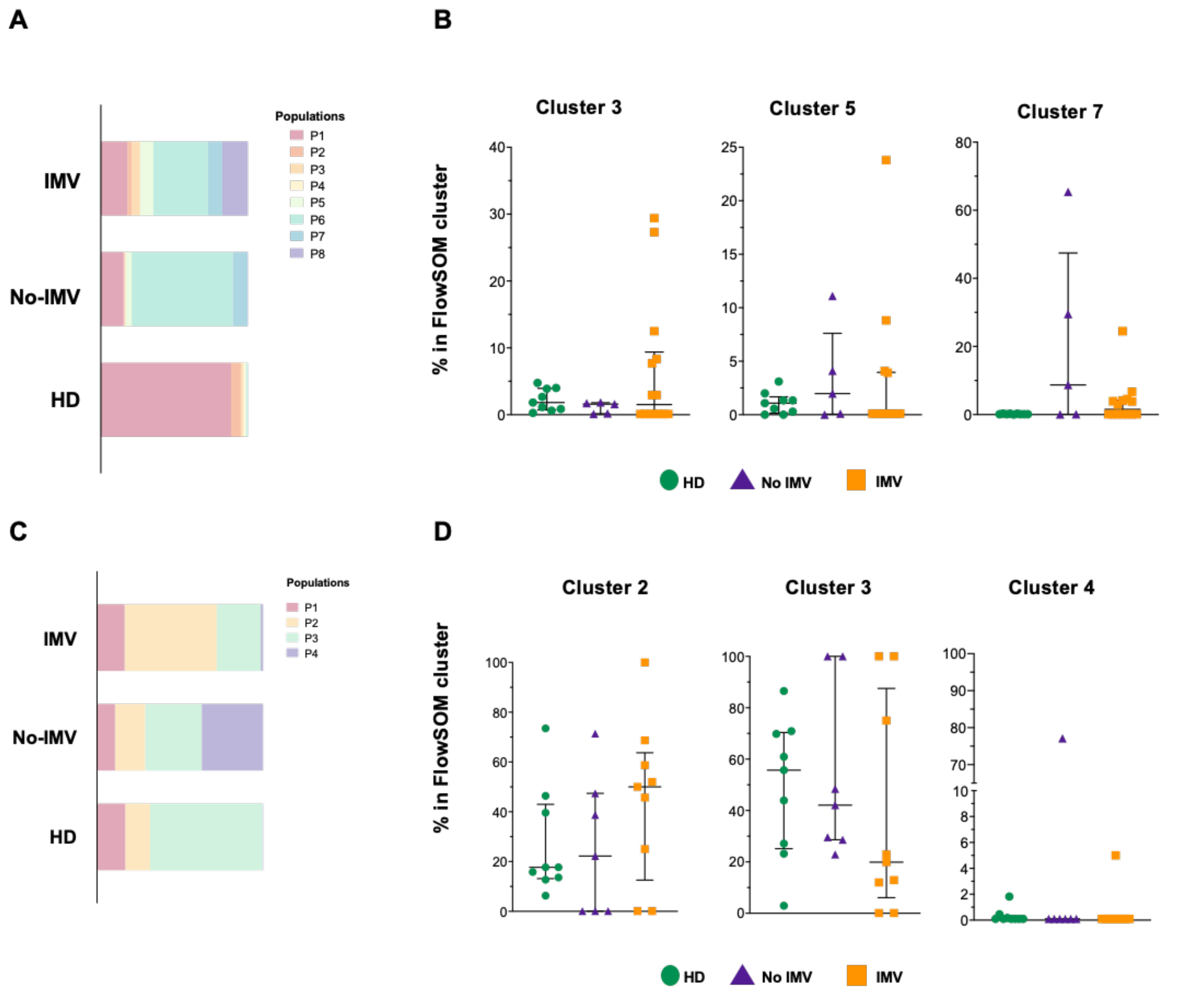

Figure S5. FlowSOM tree analysis of classical monocytes and CD3+ monocytes. For the CD14+ monocytes, the percentual distribution of all populations in each COVID-19 group and HD samples was plotted by color (A). Analysis of cluster frequencies without statistical differences between groups in CD14+ monocyte cells (B). For CD3+CD14+ monocytes, the percentual distribution of all populations in each COVID-19 group and HD samples was plotted by color. The background coloring represents the meta-clustering. Legends of the star plot and meta-clustering are shown on the right side (C). Analysis of cluster frequencies without statistical differences between groups to CD3+CD14+ monocytes (D).

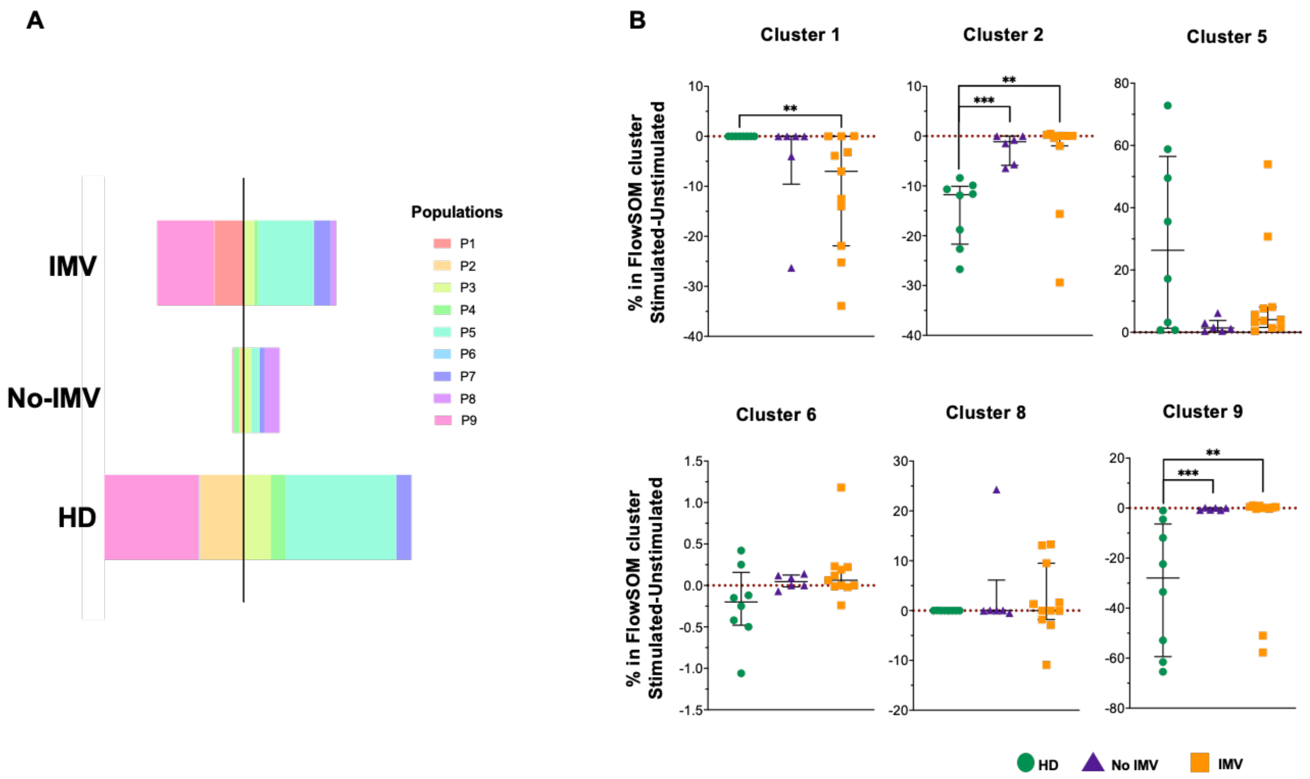

Figure S6. FlowSOM tree analysis of T-cells after the activation process. For the CD3+ T cells, the percentual distribution of all populations before (left of the black line) and after (right of the black line) polyclonal stimulus (PMA/Iono) was plotted by color. The percentual distribution of all populations in each COVID-19 group and HD samples was plotted by color (A). Analysis of cluster frequencies from T-cells after PMA/Iono stimulation, the red dotted line indicates the unstimulated level (B).
